# Demographic and work-related correlates of general and workplace loneliness among employees in Japan: a large-scale descriptive cross-sectional study

**DOI:** 10.1093/joccuh/uiaf015

**Published:** 2025-03-04

**Authors:** Norito Kawakami, Akihito Shimazu, Hisashi Eguchi, Kazuhiro Watanabe, Keiichi Matsuzaki, Reiko Inoue, Naoki Kikuchi, Yasuhiro Sekine, Akizumi Tsutsumi

**Affiliations:** Department of Digital Mental Health, Graduate School of Medicine, The University of Tokyo, Bunkyo-ku, Tokyo, Japan; Faculty of Policy Management, Keio University, Fujisawa, Kanagawa, Japan; Department of Mental Health, Institute of Industrial Ecological Sciences, University of Occupational and Environmental Health, Japan, Kitakyushu, Fukuoka, Japan; Department of Public Health, Kitasato University School of Medicine, Sagamihara, Kanagawa, Japan; Department of Public Health, Kitasato University School of Medicine, Sagamihara, Kanagawa, Japan; Kitasato University Graduate School of Medical Sciences, Sagamihara, Kanagawa, Japan; Kitasato University Graduate School of Medical Sciences, Sagamihara, Kanagawa, Japan; Kitasato University Graduate School of Medical Sciences, Sagamihara, Kanagawa, Japan; Department of Public Health, Kitasato University School of Medicine, Sagamihara, Kanagawa, Japan

**Keywords:** prevalence of loneliness, age, social class, industrial sectors, work hours

## Abstract

**Objectives:**

There has been limited research on demographic and work-related characteristics of general and workplace loneliness. The present descriptive cross-sectional study aimed to determine the demographic and work-related correlates of general and workplace loneliness in a general working population of Japan.

**Methods:**

We recruited 25 000 persons aged 20 years or older and employed by a company, organization, or government agency. We limited the sample to only employees for this analysis. Demographic and work-related characteristics were assessed by a self-report questionnaire. General and workplace loneliness were measured by single-item questions and dichotomized.

**Results:**

A total of 24 021 respondents were subjected for the analysis. Prevalences of general and workplace loneliness were 9.0% and 8.3%, respectively. Those who responded “others” or refused to answer a gender question (possibly gender minority), were middle-aged, not married, with a low household income, working in the manufacturing sector (compared with some service sectors), and reporting long working hours were associated with both general and workplace loneliness. Middle-aged groups and work hours were significantly associated with workplace loneliness after adjusting for general loneliness.

**Conclusions:**

Common demographic and work-related correlates were found for both general and workplace loneliness. Middle age and working long hours may be factors for workplace loneliness, independent of general loneliness, in Japan.

## Teaser

Key points

What is already known on this topic:

- Demographic correlates of general loneliness have been studied extensively; however, work-related correlates of general and workplace loneliness are not well known.

What this study adds:

- The study reported prevalences of both general and workplace loneliness and found common demographic and work-related correlates in a large sample of employees in Japan.
- Middle age and working hours were associated with workplace loneliness independently of general loneliness.

How this study might affect research, practice, or policy:

- The study identified groups of employees at high risk of general and workplace loneliness, which could help to understand the mechanisms and develop interventions at work.

## 1. Introduction

Loneliness, a distressing feeling of being alone, has recently attracted attention in the field of public health.^1^ Loneliness in life (general loneliness) is common and known to have a detrimental effect on people’s health and well-being.^2^ Loneliness is also common in the workplace (workplace loneliness).^3^ A British Red Cross survey reported that 11% of workers in the United Kingdom felt lonely at work “often” or “always.”^4^ Workplace loneliness has also been reported to have an impact on the health and well-being of employees as well as work-related outcomes such as burnout, lower job satisfaction, reduced work performance,^5^ and higher job turnover.^6^ Thus, general and workplace loneliness may be important determinants of employee health and productivity.

Sociodemographic correlates of general loneliness have been examined in many previous studies.^7^ Having a spouse or partner is consistently associated with lower levels of loneliness. A negative relationship between a better financial situation and loneliness has been confirmed by many cross-sectional studies. On the other hand, the difference in loneliness between men and women is often small, and the relationship between age and general loneliness is inconsistent.^7^ Some studies have reported a linear relationship, with lower levels of loneliness in younger age groups, whereas others have reported a U-shaped relationship, with higher levels of loneliness in younger and older groups. The relationship between educational attainment and general loneliness has also been inconsistent: in some studies of the general population, higher levels of education were negatively associated with loneliness, but not in others.^7^ In the general population of Japan, younger age, male gender, low income and unemployment were associated with loneliness.^8^ A nationwide survey of the general population in Japan reported that loneliness was more prevalent among men, people aged 20-59 years, those who were never married, those who lived alone, and those with an annual household income of 1 million Japanese yen (JPY) or less.^9^ In older Japanese populations, male sex, being single, and having no children were associated with general loneliness in 50-70-year-olds^10^; younger age (65-69 years), male sex, lower education, and low household income were associated with general loneliness in people over 65 years of age in Japan.^11^

Whereas many studies have reported that unemployment is associated with general loneliness,^7^ there has been limited research on the association between work-related demographic characteristics and general loneliness. One study reported that temporary workers were lonelier than permanent workers.^12^ However, other studies reported that there was little difference between workers with permanent and nonpermanent contracts.^4^^,^^8^^,^^9^ In the United Kingdom, senior managers were reported to experience more general loneliness.^4^ One study reported that working hours were associated with general loneliness among employees.^13^

Demographic or work-related correlates of workplace loneliness have been reported in far fewer studies. In one study, workplace loneliness was found to be higher among young workers (under 30 years) and lower among older workers over 50 years.^14^ Workplace loneliness was also higher among singles.^14^ The British Red Cross study found that younger age, ethnic minority status, and higher managerial position were associated with workplace loneliness.^4^ However, the study found no difference in workplace loneliness among groups classified by gender, full-time or part-time employment, industry, working from home, or household income.^4^

Investigating the demographic correlates of general and workplace loneliness is important to understand the mechanisms of loneliness development and to identify high-risk groups for effective interventions. A limited number of studies have been conducted, but most findings have not been replicated. The correlates of loneliness may vary between countries. There may be a unique pattern of correlates in a particular country. It is also interesting to know if there are unique correlates specific to general and workplace loneliness. The present large-scale descriptive study aimed to determine the demographic correlates and work-related correlates of general and workplace loneliness in a general working population of Japan. Because general and workplace loneliness strongly correlated with each other,^15^ general loneliness may confound the association between correlates and workplace loneliness, making it difficult to find unique correlates of workplace loneliness. Therefore, this study examined correlates of workplace loneliness after controlling for general loneliness. An additional analysis of correlates of respondents who suffered both from general and workplace loneliness, probably most high-risk loneliness cases, was also made.

## 2. Methods

### 2.1. Study design and subjects

This descriptive, cross-sectional study analyzed data from a large sample of employees in Japan. We recruited 25 000 individuals aged 20 years or older who were employed by a company, organization, or government agency, through an internet survey company, and asked them to complete an online questionnaire in October 2024. A further question was used to identify ineligible respondents: the self-employed, their family employees, business owners and homemakers, students, and the unemployed. We excluded these respondents. The research ethics committees of the Kitasato University Medical Ethics Organization (B24-065) and the Graduate School of Medicine/Faculty of Medicine, University of Tokyo (2024292NIe) approved this study.

### 2.2. Measurements

#### 2.2.1. General and workplace loneliness

To determine the prevalence of loneliness in general, participants were asked a single question, “How often do you feel lonely?”, which is the question to ask about loneliness recommended by the UK Office of National Statistics.^16^ However, the response options were changed from 5 in the original question to 4 (“almost never,” “occasionally,” “often,” “almost always”) to fit the questionnaire format. Those who answered “almost always” were classified as having general loneliness. Similarly, to determine the prevalence of workplace loneliness, participants were asked a single question, “How often do you feel lonely at work?”, as used in the British Red Cross Survey of Loneliness at Work,^4^ but also with the same 4-point response option. A preliminary study showed that the score (1-4) of this scale correlated moderately with the Loneliness at Work Scale (*r* = 0.507),^3^ depression/anxiety (*r* = 0.459), and mental well-being (*r* = −0.261) (Shimazu A., et al, 2024, unpublished). Those who answered “almost always” were classified as having workplace loneliness.

#### 2.2.2. Demographic and work-related characteristics

Demographic variables included gender, age, marital status, and presence of child(ren). Gender was ascertained with a 4-item response option: “male,” “female,” “other,” and “don’t want to answer.” The responses “other” and “don’t want to answer“ were combined into the category “other/refused.” Age was grouped into 5 categories: 20-29, 30-39, 40-49, 50-59, and 60+. Marital status was ascertained as married or unmarried. Respondents were asked if they had 1 or more children.

Work-related characteristics included industry, employment contract, occupation, and hours worked. Industry was defined using 20 categories then classified into 17 categories, with 3 categories of primary industries combined into 1 (agriculture, forestry, fishing and mining) due to the relatively small number of respondents for these categories (less than 100). Employment contract was established by asking whether they worked on a regular contract or nonregular contact (fixed-term, part-time, or temporary). Occupation was divided into 3 categories: managerial, nonmanual, and manual/other. Working hours per week were ascertained using 5 categories: 30 or less, 31-40, 41-50, 51-60, and 61+.

Educational attainment and household income were also established as indicators of social class. Educational attainment was defined using 8 response categories and classified into 4 categories: high-school graduates, some college graduates, university graduates or higher, and others (including junior high-school graduates). Household income was ascertained using a default question provided by the survey company scale with 7 categories and classified into 400 × 10^4^ JPY or less, 401-600 × 10^4^ JPY, 601-800 × 10^4^ JPY, 801-1000 × 10^4^ JPY, and 1001 × 10^4^ JPY or more. The median and average household incomes in Japan in 2023 were about 4 050 000 JPY and 5 240 000 JPY, respectively.

#### 2.2.3. Statistical analysis

The prevalences of general loneliness and workplace loneliness were tabulated and compared between groups classified on the basis of demographic and work-related characteristics, using the chi-squared test for significant group differences. Multiple logistic regression analysis of general loneliness or workplace loneliness was performed on the demographic and work-related characteristics. An additional multiple logistic regression analysis of workplace loneliness was conducted on the demographic and work-related characteristics, additionally adjusted for general loneliness to reveal unique correlates of workplace loneliness. In addition, a multiple logistic regression analysis of “double loneliness” (respondents having both general and workplace loneliness) was conducted in a similar manner. The level of significance was set at *P* < .05.

## 3. Results

The final sample for analysis consisted of 24 021 respondents. The majority of the sample were men, with a smaller group not responding or refusing to respond to the gender question. There was a relatively smaller number of respondents in the younger age group (20-29 years old), a larger number of nonmanual workers, and a large sample of groups with household incomes above the national average.

The prevalence of general loneliness and workplace loneliness was 9.0% (*n* = 2172) and 8.3% (*n* = 1984), respectively (Table 1). The proportion of respondents reporting both general and workplace loneliness was 6.6% (*n* = 1597); 73.5% of respondents reporting general loneliness also reported workplace loneliness; 80.5% of respondents reporting workplace loneliness also reported general loneliness. The concordance was 96.0% with k = 0.747 (*P* < .001).

**Table 1:** Prevalences (%) of general loneliness and workplace loneliness in groups classified based on demographic and work-related characteristics in a large sample of employees in Japan (*N* = 24 021).

			**General loneliness**			**Workplace loneliness**		
		***N***	***n***	**Prevalence, %**	***P***	***n***	**Prevalence, %**	***P***
**Total**		24 021	2172	9.0%		1984	8.3	
**Gender**					<.001			<.001
**Men**		15 816	1421	9.0		1328	8.4	
**Women**		7976	705	8.8		619	7.8	
**Other/refusal**		229	46	20.1		37	16.2	
**Age group, y**					<.001			<.001
**20-29**		900	78	8.7		53	5.9	
**30-39**		2979	297	10.0		255	8.6	
**40-49**		6516	635	9.7		595	9.1	
**50-59**		9386	893	9.5		837	8.9	
**60+**		4240	269	6.3		244	5.8	
**Marital status**					<.001			<.001
**Married**		15 048	1070	7.1		1041	6.9	
**Not married**		8973	1102	12.3		943	10.5	
**Child(ren)**					<.001			<.001
**Any**		13 441	990	7.4		951	7.1	
**None**		10 580	1182	11.2		1033	9.8	
**Educational attainment**					<.001			.035
**High-school graduate**		6030	569	9.4		517	8.6	
**Some college**		5038	461	9.2		407	8.1	
**University graduate or higher**		12 529	1081	8.6		1010	9.8	
**Others**		424	61	14.4		50	11.8	
**Household income (×10 000 JPY)**					<.001			<.001
**≤400**		6050	776	12.8		655	10.8	
**401-600**		5801	499	8.6%		457	7.9%	
**601-800**		5058	416	8.2		393	7.8	
**801-1000**		3403	240	7.1		232	6.8	
**1001+**		3709	241	6.5		247	6.7	
**Industrial sector**					<.001			.006
**Manufacturing**		5115	488	9.5		459	9.0	
**Agriculture, forestry, fisheries, and mining**		96	9	9.4		10	10.4	
**Construction**		1142	111	9.7		103	9.0	
**Electricity, gas, heat supply, and water**		372	39	10.5		33	8.9	
**Information and communications**		1370	110	8.0		97	7.1	
**Transport and postal services**		1375	135	9.8		133	9.7	
**Wholesale and retail trade**		2349	212	9.0		170	7.2	
**Finance and insurance**		1155	93	8.1		93	8.1	
**Real estate, goods rental and leasing**		429	39	9.1		35	8.2	
**Scientific research, professional and technical services**		472	31	6.6		28	5.9	
**Accommodations, eating and drinking services**		583	57	9.8		44	7.5	
**Living-related and personal services and amusement services**		419	37	8.8		33	7.9	
**Education, learning support**		1298	93	7.2		91	7.0	
**Medical, health care, and welfare**		2853	238	8.3		225	7.9	
**Compound services**		289	24	8.3		23	8.0	
**Services, nec.**		2072	199	9.6		172	8.3	
**Civil services, nec**		1808	824	7.7		824	7.7	
**Others**		824	118	14.3		96	11.7	
**Employment contract**					.322			.180
**Regular**		17 022	1519	8.9		1432	8.4	
**Nonregular (fixed-term, part-time, dispatched)**		6999	653	9.3		552	7.9	

**Table 1:** Continued.

			**General loneliness**			**Workplace loneliness**		
		***N***	***n***	**Prevalence, %**	***P***	***n***	**Prevalence, %**	***P***
**Occupation**					<.001			.017
**Manager**		1808	120	6.6		128	7.1	
**Nonmanual**		20 583	1870	9.1		1697	8.2	
**Manual/other**		1630	182	11.2		159	9.8	
**Work hours (per week)**					<.001			<.001
**≤30**		4020	341	8.5		278	6.9	
**31-40**		7763	694	8.9		645	8.3	
**41-50**		8375	695	8.3		633	7.6	
**51-60**		2478	213	8.6		209	8.4	
**61+**		1385	229	16.5		219	15.8	

Abbreviations: JPY, Japanese yen; nec., not elsewhere classified.

General loneliness was significantly more prevalent in the group that reported their gender as “other” or refused to answer, and there was a small difference between men and women (Table 1). General loneliness was more prevalent in groups aged 30-59 years, with a lower prevalence in younger (20-29 years) and 60+ age groups, among those who were not married, had no child, were in the “other” category of educational attainment, and had the lowest household income category (4 million JPY per year). General loneliness was more prevalent in the primary and secondary industrial sectors (than in the service sectors), in manual and other occupations, and in the group working 61+ hours per week. Demographic and work-related characteristics associated with workplace loneliness were similar to those for general loneliness, and workplace loneliness was most prevalent in the group aged 40-49 years.

In multiple logistic regression analysis, the “other/refused” category for gender, age groups 30-59, not being married, and being in the lowest household income category were significantly and positively associated with general loneliness (Table 2). Employment in information/communications, wholesale and retail trade, scientific research, professional and technical services, and education was negatively associated with general loneliness, whereas working more than 61 hours per week was positively associated with general loneliness. In the similar multiple logistic regression analysis of workplace loneliness, women had a significantly lower prevalence of workplace loneliness, whereas the “other/refused” category had a significantly higher prevalence. Age groups 30-59 years, not being married, and being in the lowest household income category were significantly and positively associated with workplace loneliness. Employment in the sectors of scientific research, professional and technical services, and education, training, medical, health care, and social assistance was negatively associated with workplace loneliness, whereas employment in the other sectors was positively associated with workplace loneliness. Working 61+ hours per week was positively associated with workplace loneliness. These patterns were similar for men and women; the analysis was not performed for those who responded “other” or refused to answer about gender, because of the small sample size.

**Table 2:** Associations of demographic and work-related characteristics with general and workplace loneliness in a large sample of employees in Japan (*N* = 24 021): odds ratios (ORs) and 95% CIs estimated by multiple logistic regressions.

		**General loneliness**					**Workplace loneliness**				
		**OR**	**95% CI**		***P***		**OR**	**95% CI**		***P***	
**Gender**											
**Men**		1					1				
**Women**		0.92	0.81-1.03		.151		0.89	0.79-1.00		.046	*
**Other/refusal**		1.69	1.17-2.44		.005	*	1.89	1.34-2.66		<.001	*
		Wald chi-square = 11.0, *df* = 2, *P* = .004					Wald chi-square = 19.7, *df* = 2, *P* < .001				
**Age group, y**											
**20-29**		1					1				
**30-39**		1.65	1.21-2.25		<.001	*	1.31	1.01-1.71		.046	*
**40-49**		1.86	1.38-2.51		<.001	*	1.35	1.05-1.74		.020	*
**50-59**		1.92	1.42-2.58		<.001	*	1.41	1.09-1.82		.008	*
**60+**		1.27	0.92-1.76		.150		0.96	0.72-1.27		.768	
		Wald chi-square = 45.8, *df* = 4, *P* < .001					Wald chi-square = 31.9, *df* = 4, *P* < .001				
**Marital status**											
**Married**		1					1				
**Not married**		1.33	1.17-1.52		<.001	*	1.47	1.30-1.67		<.001	*
**Child(ren)**											
**Any**		1					1				
**None**		1.09	0.96-1.23		.194		1.08	0.96-1.22		.204	
**Educational attainment**											
**High-school graduate**		1									
**Some college**		1.00	0.87-1.15		.961		1.04	0.91-1.19		.601	
**University graduate or higher**		1.06	0.94-1.20		.339		1.08	0.96-1.21		.223	
**Others**		1.18	0.86-1.61		.312		1.28	0.96-1.72		.091	
		Wald chi-square = 2.1, *df* = 3, *P* = .546					Wald chi-square = 3.7, *df* = 3, *P* = .293				
**Household income (×10 000 JPY)**											
**≤400**		1					1				
**401-600**		0.72	0.63-0.82		<.001	*	0.69	0.61-0.78		<.001	*
**601-800**		0.71	0.62-0.83		<.001	*	0.68	0.59-0.79		<.001	*
**801-1000**		0.63	0.53-0.75		<.001	*	0.60	0.50-0.71		<.001	*
**1001+**		0.61	0.51-0.73		<.001	*	0.55	0.47-0.66		<.001	*
		Wald chi-square = 45.1, *df* = 4, *P* < .001					Wald chi-square = 64.6, *df* = 4, *P* < .001				
**Industrial sector**											
**Manufacturing**		1					1				
**Agriculture, forestry, fisheries, and mining**		1.15	0.59-2.25		.680		0.92	0.46-1.85		.811	
**Construction**		0.99	0.79-1.25		.961		1.02	0.82-1.27		.885	
**Electricity, gas, heat supply, and water**		1.03	0.71-1.49		.891		1.15	0.81-1.63		.421	
**Information and communications**		0.77	0.61-0.97		.026	*	0.82	0.66-1.02		.074	
**Transport and postal services**		0.95	0.77-1.17		.638		0.87	0.71-1.07		.202	
**Wholesale and retail trade**		0.77	0.64-0.93		.006	*	0.88	0.74-1.05		.163	
**Finance and insurance**		0.93	0.74-1.18		.572		0.89	0.70-1.13		.331	
**Real estate, goods rental and leasing**		0.92	0.64-1.32		.637		0.96	0.68-1.36		.823	
**Scientific research, professional and technical services**		0.63	0.42-0.93		.020	*	0.65	0.44-0.95		.025	*
**Accommodations, eating and drinking services**		0.80	0.58-1.11		.183		0.93	0.69-1.25		.634	
**Living-related and personal services and amusement services**		0.84	0.58-1.22		.369		0.84	0.59-1.20		.345	
**Education, learning support**		0.76	0.59-0.96		.023	*	0.71	0.56-0.90		.005	*
**Medical, health care, and welfare**		0.88	0.74-1.04		.141		0.84	0.71-0.99		.043	*
**Compound services**		0.89	0.57-1.39		.609		0.85	0.55-1.30		.446	
**Services, nec.**		0.87	0.72-1.05		.155		0.91	0.76-1.09		.299	
**Civil services, nec**		0.91	0.74-1.11		.348		0.86	0.70-1.05		.127	
**Others**		1.21	0.95-1.53		.128		1.33	1.07-1.67		.012	*
		Wald chi-square = 25.9, *df* = 17, *P* = .076					Wald chi-square = 32.1, *df* = 17, *P* = .014				
**Employment contract**											
**Regular contract**											
**Nonregular (fixed-term, part-time, dispatched)**		0.97	0.85-1.10		.611		1.01	0.89-1.15		.838	

**Table 2:** Continued.

		**General loneliness**					**Workplace loneliness**				
		**OR**	**95% CI**		***P***		**OR**	**95% CI**		***P***	
**Occupation**											
**Manager**		1.00									
**Nonmanual**		1.09	0.90-1.33		.378		1.21	0.99-1.48		.069	
**Manual/other**		1.05	0.81-1.36		.694		1.23	0.96-1.59		.108	
		Wald chi-square = 0.9, *df* = 2, *P* = .643					Wald chi-square = 3.5, *df* = 2, *P* = .177				
**Work hours (per week)**											
**≤30**		1									
**31-40**		1.16	0.99-1.36		.065		1.03	0.89-1.19		.678	
**41-50**		1.06	0.89-1.25		.518		0.98	0.84-1.15		.836	
**51-60**		1.22	0.99-1.50		.064		1.08	0.88-1.32		.470	
**61+**		2.47	2.00-3.05		<.001	*	2.25	1.84-2.76		<.001	*
		Wald chi-square = 105.5, *df*= 4, *P*< .001					Wald chi-square = 102.9, *df* = 4, *P* < .001				

Abbreviations: JPY, Japanese yen; nec., not elsewhere classified.

^*^  *P* < .05.

When general loneliness was additionally adjusted for in the multiple logistic regression analysis of workplace loneliness, only age groups and hours worked per week were significantly associated with workplace loneliness. Odds ratios (ORs) (95% CI) were 1.73 (1.12-2.66) for ages 30-39, 2.14 (1.42-3.24) for ages 40-49, 2.13 (2.13-1.41) for ages 50-59, and 1.65 (1.05-2.58) for ages 60+, compared with ages 20-29. ORs (95% CI) associated with hours worked per week were 1.30 (1.04-1.64) for 31-40 hours; 1.15 (0.90-1.48) for 41-50 hours; 1.37 (1.00-1.86) for 51-60 hours; and 1.88 (1.35-2.62) for 61+ hours.

The multiple logistic regression analysis of “double loneliness” (respondents having both general and workplace loneliness) indicated a similar pattern for each of general or workplace loneliness (Tables S1 and S2). Women had a significantly lower prevalence of workplace loneliness, whereas the “other/refused” category had a significantly higher prevalence. Age groups 30 years or older, not being married, and being in the lowest household income category were significantly and positively associated with double loneliness. Employment in some secondary sectors (ie, information and communications; wholesale and retail trade; and education, learning support) was negatively associated with double loneliness. Working 61+ hours per week was positively associated with double loneliness.

## 4. Discussion

In this descriptive study, gender (the “other/refused” category), age, marital status, household income, industry, and working hours were associated with general loneliness and workplace loneliness, as well as “double loneliness” (having both types of loneliness). In addition, women had a significantly lower risk of workplace loneliness than men. Age and working hours were uniquely and significantly associated with workplace loneliness after adjusting for general loneliness.

The prevalence of general and workplace loneliness among Japanese employees in this study was 9.0% and 8.3%, respectively. These prevalences are close to the prevalence of general and workplace loneliness in the United Kingdom (13% and 11%, respectively).^4^ The prevalence of general loneliness was higher than that reported among workers in a nationwide survey in Japan (5.0%-7.3%).^9^ It should be noted that there were different methodologies used to estimate general and workplace loneliness. The present study used a 4-point scale, defining those who responded “almost always” as being lonely, whereas the other surveys used a 5-point scale, defining those who responded “often or always” as being lonely.^4^^,^^9^ The present scale and the definition may over- or under-estimate the prevalence. Further efforts are required to develop a standard assessment of loneliness and calibrate these scales against it to estimate a true prevalence.

Although a high concordance was observed between general and work loneliness, 26.5% of respondents who had general loneliness reported no work loneliness; 19.5% of respondents who had work loneliness reported no general loneliness. This is consistent with a previous observation that general loneliness and workplace loneliness are not perfectly aligned (*r* = 0.695).^15^ These findings support the view that work loneliness is a distinct construct in the work environment, separate from general loneliness,^3^ although there is a strong overlap.

The present study found that some demographic characteristics were commonly associated with both general loneliness and workplace loneliness. Some of the findings were consistent with previous studies of general loneliness: marital status and low household income.^6^ A new finding was that people who rated their gender as “other” or refused to answer about gender were lonelier in general life and at work. Although we did not ask whether these respondents were LGBTQ, it is likely that gender minorities had a higher prevalence of general and workplace loneliness. Japan does not have a cultural history of strong stigma against homosexuality and gender nonconformity.^17^ However, sexual minorities may face pervasive discrimination and even bullying, and disclosure as belonging to a sexual minority is often difficult in the community and workplace.^17^ This could lead to general and workplace loneliness in this group. Women had a significantly lower prevalence than men for workplace loneliness. A similar pattern was also found for general loneliness, but it was nonsignificant. There have been only small sex or gender differences in general loneliness reported in the previous literature.^6^ Men may be more committed to their work and thus more affected by workplace social relationships and subject to feelings of loneliness than women in a gender-segregated culture like Japan.^18^ The pattern may also be attributed to the fact that men tend to have fewer social relationships outside a romantic/marital relationship than women do.^19^ The middle-aged had higher prevalences of general and workplace loneliness. This age pattern was similar to the national survey of general loneliness in Japan.^11^ Loneliness may be more prevalent in the working-age population. This may be due to a relatively smaller social network among middle-aged people in Japan.^20^ In middle age, the quantity and quality of the social network may decrease due to the demands and responsibilities of work and family life, which may lead to increased loneliness. The observed age pattern differed from the pattern of younger age groups being lonelier as found in previous studies^7^ and in a study in Japan.^8^ This may be because the present sample did not include students, who tend to be lonely.^9^

Among the work-related characteristics examined in this study, several service sectors had a lower prevalence of general and workplace loneliness compared with manufacturing. Service sector employment may involve more social interactions at work, such as with members and customers or clients, which could lead to a lower prevalence of both workplace and general loneliness. Working more than 61 hours per week was also significantly associated with general and workplace loneliness. This is consistent with a previous observation that work hours are associated with general loneliness among workers.^13^ A qualitative study also reported that workload may prevent opportunities for social interactions at work, for example, by discouraging meetings.^5^ Another possibility is that long working hours may worsen mental health,^21^ which in turn may increase feelings of loneliness.^7^ A previous report in the United Kingdom found that higher managerial positions were associated with workplace loneliness.^4^ This was not replicated in the present study. The present study found an association between low household income and workplace loneliness, which was not observed in a previous report in the United Kingdom.^4^ Japan is known to be a more equal society and have higher social cohesion than the United Kingdom.^22^ Managers in Japan may be more likely to maintain companionship with subordinates despite their greater responsibility, compared with their counterparts in the United Kingdom.^18^ A previous report also suggested that the relationship between income and well-being is stronger in more equal societies, because a given level of income reduction in such societies may be perceived as a greater reduction in social position.^23^ Hence, the relationship between income and workplace isolation may be more marked in Japan. It may also be attributable to a greater deterioration of well-being in the low-income group during the COVID-19 pandemic in Japan.^24^

Middle age and working hours were associated with workplace loneliness independently of general loneliness. Although these variables were found to be associated with general loneliness, they may uniquely contribute also to workplace loneliness. This suggests a link between the work environment and workplace loneliness beyond general loneliness. Workers may become more socialized to the workplace as they age. The quantity and quality of interpersonal relationships at work may become more important to middle-aged workers, and their expectations for social interactions may be greater as they age. Since workplace loneliness is caused by a mismatch between actual and expected social relationships at work,^25^ middle-aged workers may be more likely to experience workplace loneliness due to their high expectations of social relationships at work. This may be particularly true in Japan, a country that still maintains a collective workplace culture.^18^ Working 61+ hours per week was significantly associated with general loneliness, workplace loneliness, and double loneliness. Working 61+ hours per week was associated with workplace loneliness independently of general loneliness, suggesting that working hours play a role in the job-specific mechanisms of developing workplace loneliness. As discussed earlier, long working hours may interfere with social interactions at work.^5^ Employees who work longer than their colleagues may perceive this as unequal and frustrating, leading to feelings of alienation. Deteriorated social relationships at work due to long working hours should be investigated as a possible mechanism of workplace loneliness.

### 4.1. Limitations

The present sample was recruited from a pooled larger sample of an internet survey company. The sample was biased, with a lower proportion of younger age groups, a higher proportion of highly educated and high household income groups, and of nonmanual workers compared with national statistics of Japan. The present sample did not include other types of workers such as the self-employed, informal sector workers, or individuals working in the gig economy. The prevalence of loneliness should be investigated in these populations. The single-item 4-point scales of general and workplace loneliness used in this study were not fully validated. A single-item direct question about loneliness may introduce a bias to underreport loneliness because of the stigma associated with loneliness. However, a report suggested that the bias may not be substantial.^26^ Some of the demographic and work-related characteristics should have been asked about in a more explicit way: gender minority was not questioned directly; also, employment contracts were questioned with dichotomized response categories that did not classify nonregular contracts as fixed-term, part-time, or temporary. In addition, we did not ask respondents for some important factors, such as working from home, which has been reported as a risk factor of general loneliness.^27^

## 5. Conclusion

The prevalence of general and workplace loneliness among Japanese employees in this study was 9.0% and 8.3%, respectively, in the present descriptive cross-sectional study of a large sample of employees in Japan. The study found that answering “other” and refusing a gender question (possibly gender minority), being middle-aged, not being married, having a low household income, being in manufacturing (compared with some service sectors), and working long hours were associated with both general and workplace loneliness. Being middle-aged and working long hours were more associated with workplace loneliness than with general loneliness. Employees who are middle-aged and work long hours may be a high-risk group for workplace loneliness, and could be a target for workplace interventions to prevent and mitigate workplace loneliness.

## Supplementary Material

Web_Material_uiaf015

## Data Availability

The data supporting this study’s findings are available from the corresponding author upon reasonable request.
