# Supplementary material for: Demographic and work-related correlates of general and workplace loneliness among employees in Japan: a large-scale descriptive cross-sectional study
Source: J Occup Health. 2025 Mar 4;67(1):uiaf015. doi: 10.1093/joccuh/uiaf015 (PMC11931285; doi:10.1093/joccuh/uiaf015)
Supplement: Web_Material_uiaf015 [file web_material_uiaf015.zip › DemoLonely_STables_JOHformat_250215.docx]

Supplementary Table S1 - Prevalences (%) of "double loneliness" (having both general and workplace loneliness) * in groups classified based on demographic and work-related characteristics in a large sample of employees in Japan (N=24,021)

|  |  |  | "Double loneliness"* | | |  |
| --- | --- | --- | --- | --- | --- | --- |
|  |  | N | n | Prevalence | p | |
| Total |  | 24,021 | 1,597 | 6.6% |  | |
| Gender |  |  |  |  | <0.001 | |
|  | Men | 15,816 | 1,079 | 6.8% |  | |
|  | Women | 7,976 | 484 | 6.1% |  | |
|  | Other/refusal | 229 | 34 | 14.8% |  | |
| Age group (years) | |  |  |  | <0.001 | |
|  | 20-29 | 900 | 38 | 4.2% |  | |
|  | 30-39 | 2,979 | 204 | 6.8% |  | |
|  | 40-49 | 6,516 | 470 | 7.2% |  | |
|  | 50-59 | 9,386 | 686 | 7.3% |  | |
|  | 60+ | 4,240 | 199 | 4.7% |  | |
| Marital status | |  |  |  | <0.001 | |
|  | Married | 15,048 | 826 | 5.5% |  | |
|  | Not married | 8,973 | 771 | 8.6% |  | |
| Child(ren) | |  |  |  | <0.001 | |
|  | Any | 13,441 | 759 | 5.6% |  | |
|  | None | 10,580 | 838 | 7.9% |  | |
| Educational attainment | |  |  |  | 0.074 | |
|  | High school graduate | 6,030 | 424 | 7.0% |  | |
|  | Some college | 5,038 | 326 | 6.5% |  | |
|  | University graduate or higher | 12,529 | 808 | 6.4% |  | |
|  | Others | 424 | 39 | 9.2% |  | |
| Household income (x 10,000 JPY) | |  |  |  | <0.001 | |
|  | <=400 | 6,050 | 543 | 9.0% |  | |
|  | 401-600 | 5,801 | 359 | 6.2% |  | |
|  | 601-800 | 5,058 | 314 | 6.2% |  | |
|  | 801-1000 | 3,403 | 190 | 5.6% |  | |
|  | 1001+ | 3,709 | 191 | 5.1% |  | |
| Industrial sector | |  |  |  | 0.006 | |
|  | Manufacturing | 5,115 | 370 | 7.2% |  | |
|  | Agriculture, forestry, fisheries, and mining | 96 | 8 | 8.3% |  | |
|  | Construction | 1,142 | 87 | 7.6% |  | |
|  | Electricity, gas, heat supply and water | 372 | 29 | 7.8% |  | |
|  | Information and communications | 1,370 | 76 | 5.5% |  | |
|  | Transport and postal services | 1,375 | 105 | 7.6% |  | |
|  | Wholesale and retail trade | 2,349 | 141 | 6.0% |  | |
|  | Finance and insurance | 1,155 | 71 | 6.1% |  | |
|  | Real estate and goods rental and leasing | 429 | 28 | 6.5% |  | |
|  | Scientific research, professional and technical services | 472 | 24 | 5.1% |  | |
|  | Accommodations, eating and drinking services | 583 | 36 | 6.2% |  | |
|  | Living-related and personal services and amusement services | 419 | 25 | 6.0% |  | |
|  | Education, learning support | 1,298 | 68 | 5.2% |  | |
|  | Medical, health care and welfare | 2,853 | 177 | 6.2% |  | |
|  | Compound services | 289 | 19 | 6.6% |  | |
|  | Services, nec. | 2,072 | 138 | 6.7% |  | |
|  | Civil services, nec | 1,808 | 113 | 6.2% |  | |
|  | Others | 824 | 82 | 10.0% |  | |
| Employment contract | |  |  |  | 0.324 | |
|  | Regular contract | 17,022 | 1,149 | 6.8% |  | |
|  | Non-regular (fixed-term, part-time, dispatched) | 6,999 | 448 | 6.4% |  | |
| Occupation | |  |  |  | 0.009 | |
|  | Managers | 1,808 | 98 | 5.4% |  | |
|  | Non-manual | 20,583 | 1,368 | 6.6% |  | |
|  | Manual/other | 1,630 | 131 | 8.0% |  | |
| Work hours (per week) | |  |  |  | <0.001 | |
|  | <=30 | 4,020 | 223 | 5.5% |  | |
|  | 31-40 | 7,763 | 531 | 6.8% |  | |
|  | 41-50 | 8,375 | 499 | 6.0% |  | |
|  | 51-60 | 2,478 | 152 | 6.1% |  | |
|  | 61+ | 1,385 | 192 | 13.9% |  | |

* Defined as having both general loneliness and workplace loneliness.

Supplementary Table S2 - Associations of demographic and work-related characteristics with "double loneliness" (having both general and workplace loneliness) in a large sample of employees in Japan (N=24,021): Odds ratios (Ors) and 95% confidence intervals (CIs) estimated by a multiple logistic regression

|  |  | "double loneliness"§ | | | |  |
| --- | --- | --- | --- | --- | --- | --- |
|  |  | OR | 95%CI | | p |  |
| Gender | |  |  |  |  |  |
|  | Men | 1 |  |  |  |  |
|  | Women | 0.87 | 0.76 | -- 0.99 | 0.042 | * |
|  | Other/refusal | 1.87 | 1.28 | -- 2.74 | 0.001 | * |
|  |  | Wald chi-square=16.5, DF=2, p<0.001 | | | |  |
| Age group (years) | |  |  |  |  |  |
|  | 20-29 | 1 |  |  |  |  |
|  | 30-39 | 1.84 | 1.28 | -- 2.63 | 0.001 | * |
|  | 40-49 | 2.04 | 1.45 | -- 2.89 | <0.001 | * |
|  | 50-59 | 2.19 | 1.55 | -- 3.10 | <0.001 | * |
|  | 60+ | 1.45 | 1.00 | -- 2.11 | 0.050 | * |
|  |  | Wald chi-square=40.9, DF=4, p<0.001 | | | |  |
| Marital status | |  |  |  |  |  |
|  | Married | 1 |  |  |  |  |
|  | Not married | 1.37 | 1.19 | -- 1.59 | <0.001 | * |
| Child(ren) | |  |  |  |  |  |
|  | Any | 1 |  |  |  |  |
|  | None | 1.09 | 0.94 | -- 1.25 | 0.249 |  |
| Educational attainment | |  |  |  |  |  |
|  | High school graduate | 1 |  |  |  |  |
|  | Some college | 0.99 | 0.85 | -- 1.16 | 0.913 |  |
|  | University graduate or higher | 1.06 | 0.93 | -- 1.21 | 0.406 |  |
|  | Others | 1.09 | 0.77 | -- 1.54 | 0.642 |  |
|  |  | Wald chi-square=1.2, DF=3, p=0.750 | | | |  |
| Household income (x 10,000 JPY) | |  |  |  |  |  |
|  | <=400 | 1 |  |  |  |  |
|  | 401-600 | 0.68 | 0.59 | -- 0.79 | <0.001 | * |
|  | 601-800 | 0.70 | 0.59 | -- 0.82 | <0.001 | * |
|  | 801-1000 | 0.63 | 0.52 | -- 0.76 | <0.001 | * |
|  | 1001+ | 0.58 | 0.47 | -- 0.71 | <0.001 | * |
|  |  | Wald chi-square=42.9, DF=4, p<0.001 | | | |  |
| Industrial sector | |  |  |  |  |  |
|  | Manufacturing | 1 |  |  |  |  |
|  | Agriculture, forestry, fisheries, and mining | 1.14 | 0.54 | -- 2.38 | 0.736 |  |
|  | Construction | 1.05 | 0.82 | -- 1.34 | 0.691 |  |
|  | Electricity, gas, heat supply and water | 1.14 | 0.77 | -- 1.69 | 0.519 |  |
|  | Information and communications | 0.75 | 0.58 | -- 0.97 | 0.031 | * |
|  | Transport and postal services | 0.91 | 0.73 | -- 1.15 | 0.448 |  |
|  | Wholesale and retail trade | 0.79 | 0.65 | -- 0.97 | 0.027 | * |
|  | Finance and insurance | 0.89 | 0.68 | -- 1.17 | 0.403 |  |
|  | Real estate and goods rental and leasing | 0.92 | 0.61 | -- 1.37 | 0.666 |  |
|  | Scientific research, professional and technical services | 0.67 | 0.44 | -- 1.03 | 0.066 |  |
|  | Accommodations, eating and drinking services | 0.82 | 0.57 | -- 1.18 | 0.289 |  |
|  | Living-related and personal services and amusement services | 0.79 | 0.52 | -- 1.21 | 0.280 |  |
|  | Education, learning support | 0.70 | 0.53 | -- 0.92 | 0.010 | * |
|  | Medical, health care and welfare | 0.87 | 0.71 | -- 1.05 | 0.150 |  |
|  | Compound services | 0.92 | 0.57 | -- 1.49 | 0.738 |  |
|  | Services, nec. | 0.86 | 0.70 | -- 1.06 | 0.165 |  |
|  | Civil services, nec | 0.91 | 0.73 | -- 1.14 | 0.423 |  |
|  | Others | 1.26 | 0.97 | -- 1.63 | 0.086 |  |
|  |  | Wald chi-square=25.9, DF=17, p=0.077 | | | |  |
| Employment contract | |  |  |  |  |  |
|  | Regular contract | 1 |  |  |  |  |
|  | Non-regular (fixed-term, part-time, dispatched) | 0.96 | 0.83 | -- 1.11 | 0.589 |  |
| Occupation | |  |  |  |  |  |
|  | Managers | 1 |  |  |  |  |
|  | Non-manual | 1.16 | 0.93 | -- 1.45 | 0.190 |  |
|  | Manual/other | 1.13 | 0.85 | -- 1.51 | 0.408 |  |
|  |  | Wald chi-square=1.7, DF=2, p=0.417 | | | |  |
| Work hours (per week) | |  |  |  |  |  |
|  | <=30 | 1 |  |  |  |  |
|  | 31-40 | 1.18 | 0.99 | -- 1.40 | 0.067 |  |
|  | 41-50 | 1.02 | 0.85 | -- 1.23 | 0.810 |  |
|  | 51-60 | 1.08 | 0.86 | -- 1.37 | 0.505 |  |
|  | 61+ | 2.66 | 2.12 | -- 3.34 | <0.001 | * |
|  |  | Wald chi-square=117.2, DF=4, p<0.001 | | | |  |

§ Defined as having both general loneliness and workplace loneliness.

* p<0.05.
